# Supplementary material for: Calmodulin enhances mTORC1 signaling by preventing TSC2-Rheb binding
Source: J Biol Chem. 2024 Dec 22;301(2):108122. doi: 10.1016/j.jbc.2024.108122 (PMC11787510; doi:10.1016/j.jbc.2024.108122)
Supplement: Supporting Information [file mmc1.pdf]

Supporting information

Calmodulin enhances mTORC1 signaling by preventing TSC2-Rheb binding.

Yuna Amemiya[1], Yuichiro Ioi[1], Makoto Araki[2], Kenji Kontani[2], Masatoshi Maki[1], Hideki Shibata[1] and Terunao Takahara[1]*

[1]Graduate School of Bioagricultural Sciences, Nagoya University, Furo-cho, Chikusa, Nagoya, Japan
[2]Department of Biochemistry, Meiji Pharmaceutical University, Tokyo, Japan

*Corresponding author

List of material included:

Figure S1……………………………………….page S-2
Figure S2……………………………………….page S-3
Figure S3……………………………………….page S-4
Figure S4……………………………………….page S-5
Figure S5……………………………………….page S-6
Figure S6……………………………………….page S-7
Figure S7……………………………………….page S-8
Figure S8……………………………………….page S-9

Legends for supplemental movies……………page S-10

As separate files:
Movie S1
Movie S2
Movie S3
Movie S4

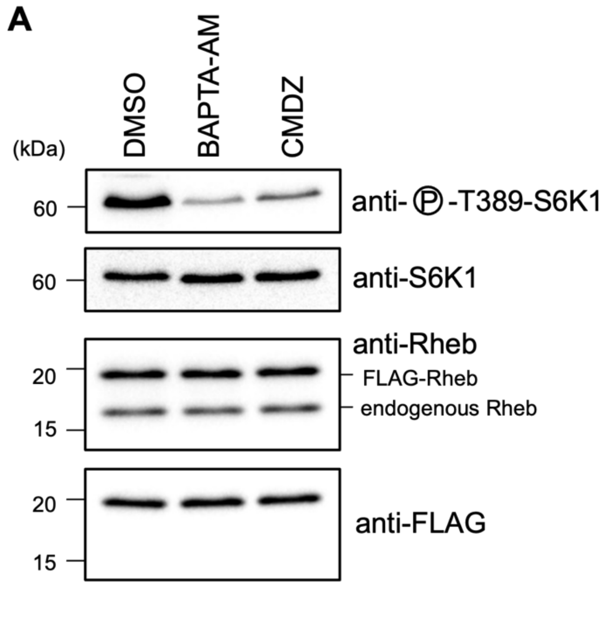

Figure S1. The level of GTP-bound Rheb was reduced following treatment with a Ca^{2+} chelator or a CaM inhibitor in cells.

(A) HEK293T cells expressing FLAG-Rheb in a doxycycline (Dox)-dependent manner was cultured in the presence of 10 ng/ml of Dox for 24 h, and treated with DMSO, BAPTA-AM (50 μM), or CMDZ (30 μM) for 30 min. Cell lysates were subjected to Western blot analysis using the indicated antibodies.

(B) Immunoprecipitates using the anti-FLAG antibody from the cell lysates in (A) were subjected to Fluor-HPLC analysis, and the relative amounts of guanine nucleotides associated with FLAG-Rheb were quantified from the peak areas of GTP and GDP. Data represent mean ± SD from three independent experiments. One-way ANOVA with Tukey's test, $^*p < 0.05$.

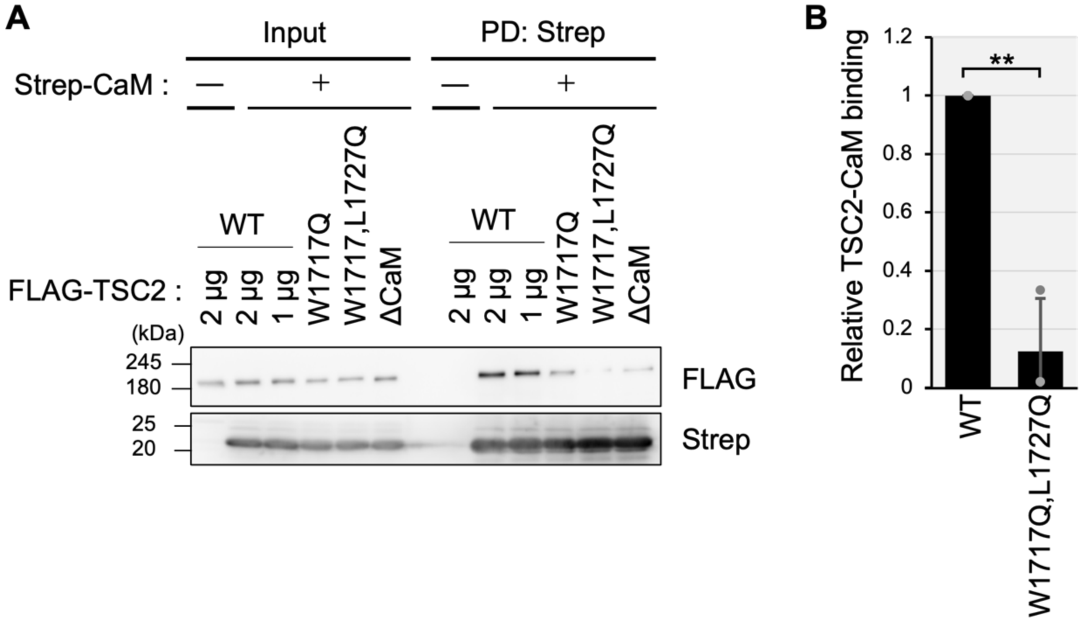

Figure S2. The TSC2 mutant with W1717Q and L1727Q fails to bind to CaM.

(A) HEK293T cells were transiently transfected with plasmids encoding FLAG-TSC2 wild type (WT) or mutants (W1717Q, W1717Q and L1727Q, or ΔCaM) together with the plasmid for Strep-CaM for 24 h. In FLAG-TSC2 WT, different amounts of the plasmid were used for transfection. Cell lysates were subjected to pulldown with Streptavidin beads and analyzed by Western blotting with anti-FLAG and anti-Strep antibodies.

(B) Graphs represent mean ± SD of three independent experiments, in which relative binding of FLAG-TSC2 WT to Strep-CaM was set to 1. One-way ANOVA with Tukey's test, ***p* < 0.01.

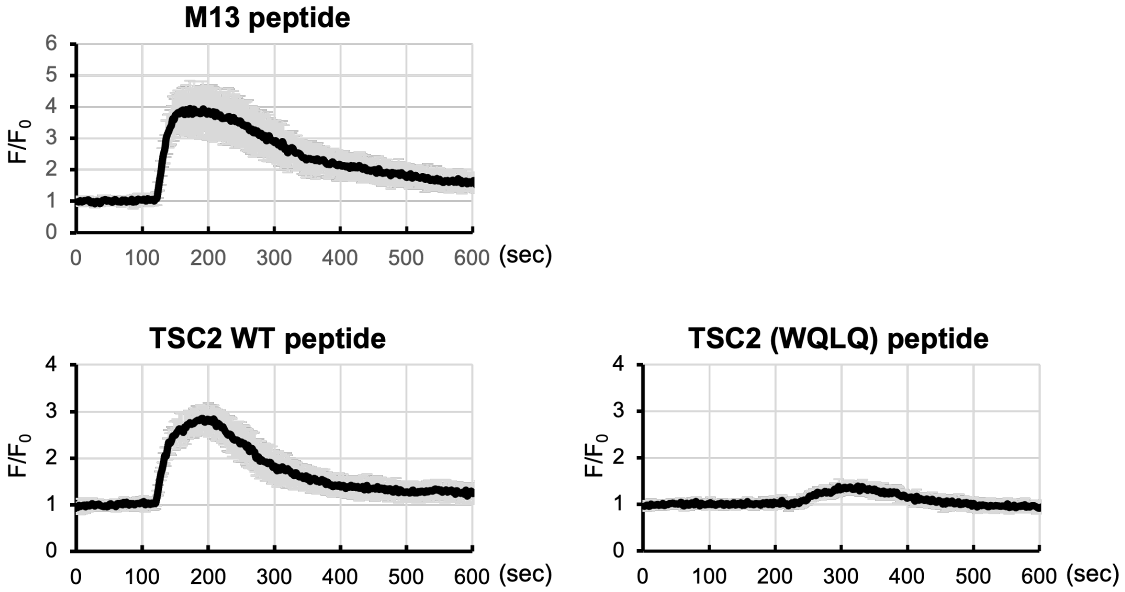

Figure S3. The addition of amino acids increases the fluorescence signal of R-GECO1-TSC2 probe.

HEK293 cells were transiently transfected with the plasmid encoding R-GECO1(M13 peptide), R-GECO1(TSC2 WT peptide) or R-GECO1(TSC2 (WQLQ) peptide). The medium was replaced with HBSS for 60 min, and then amino acids mixture was added at 120 s. The ratio of fluorescence signals for R-GECO1 to the average signals before the addition of amino acids mixture (F_0) was shown. Graphs are shown as mean ± SD (n = 15).

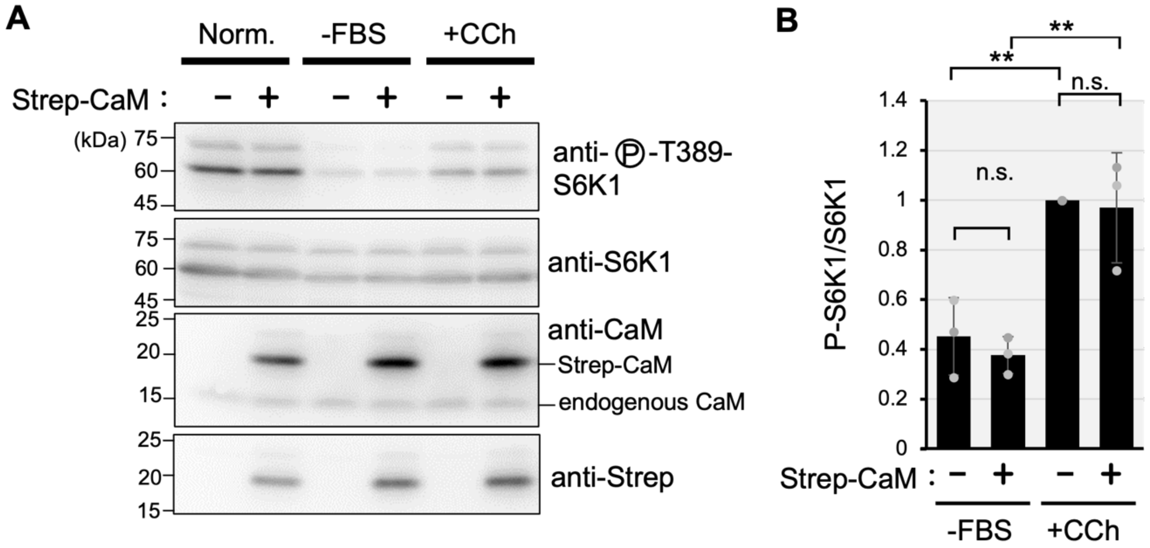

Figure S4. Overexpression of CaM does not affect carbachol-induced mTORC1 activation.

(A) HEK293 cells stably expressing Strep-CaM (+) or control (-) were cultured in the normal media (Norm.) or starved of serum (−FBS) for 3 h, and then stimulated with carbachol (+CCh,100 μM) for 15 min. Cell lysates were analyzed by Western blotting with indicated antibodies.

(B) Quantitation of the relative intensity of phospho-Thr389-S6K1 to total S6K1 of –FBS and +CCh in (A), in which the +CCh condition in Ctrl cells was set to 1. Graphs represent mean ± SD of three independent experiments. One-way ANOVA with Tukey's test, $^{**}p < 0.01$, n.s.; not significant.

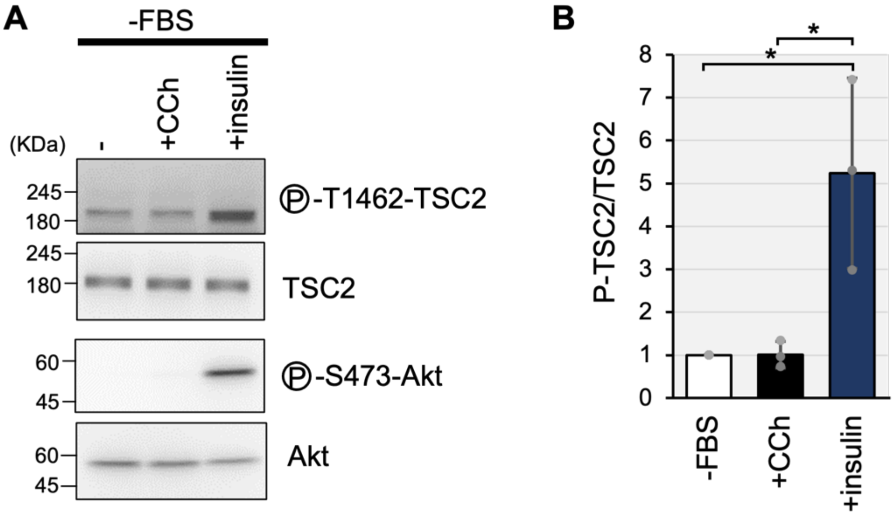

Figure S5. Carbachol does not affect Akt-mediated phosphorylation of TSC2 at Thr1462.

(A) HEK293 cells were starved of serum (−FBS) for 3 h, and then stimulated with carbachol (+CCh,100 μM) or insulin (100 nM) for 15 min. Cell lysates were analyzed by Western blotting with the indicated antibodies.

(B) Quantitation of the relative intensity of phospho-Thr1462-TSC2 to total TSC2 of (A), in which the –FBS condition was set to 1. Graphs represent mean ± SD of three independent experiments. One-way ANOVA with Tukey's test, *$p < 0.05$.

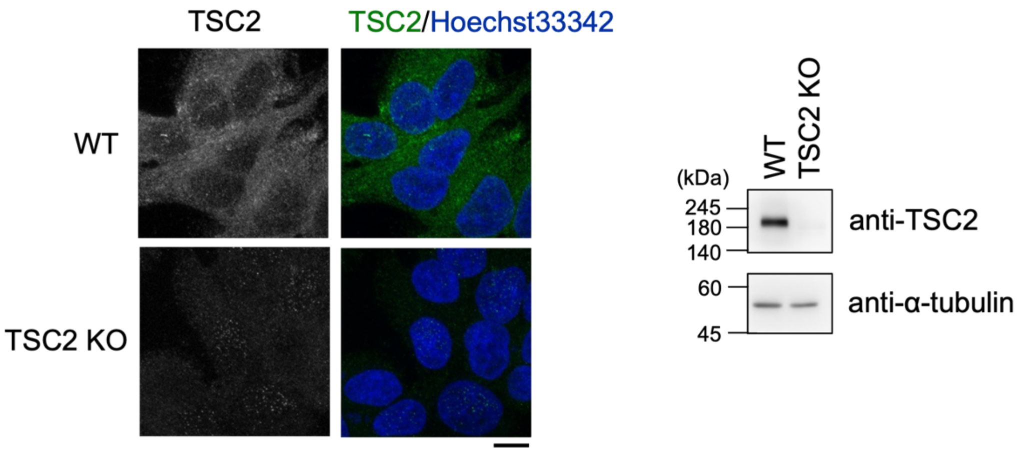

Figure S6. Validation of the TSC2 antibody used in immunofluorescence experiments.

HEK293 cells wild type (WT) and HEK293 TSC2 KO cells (TSC2 KO) were starved of serum for 3 h. The cells were then immunostained with the anti-TSC2 antibody. Merged images of TSC2 (green) and nuclei staining with Hoechst 33342 (blue) are shown. Scale bar, 10 µm. Cell lysates from WT and TSC2 KO cells were also analyzed by Western blotting with indicated antibodies.

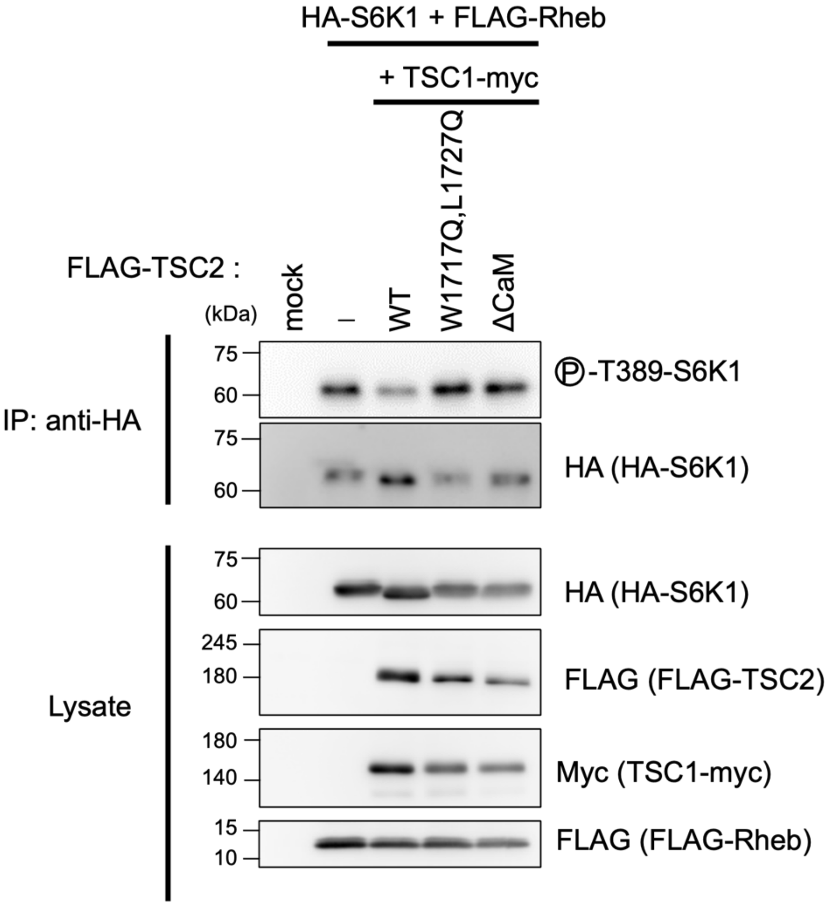

Figure S7. TSC2 mutants lack GAP activity toward Rheb.

HEK293T cells were transiently transfected without any plasmid (mock) or with plasmids encoding HA-S6K1 and FLAG-Rheb, together with plasmids for TSC1-myc and FLAG-TSC2 variants as indicated. After 24 h of transfection, cell lysates were recovered and subjected to immunoprecipitation (IP) with the anti-HA antibody. Both the immunoprecipitates and cell lysates were analyzed by Western blotting with indicated antibodies.

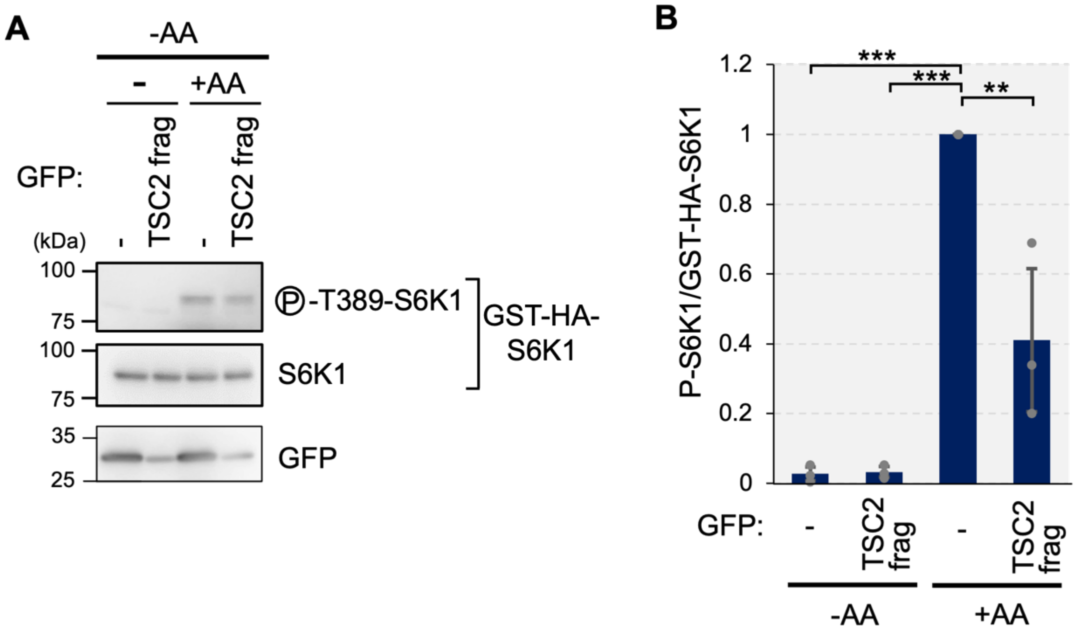

Figure S8. Overexpression of the CaM-binding region of TSC2 prevents S6K1 phosphorylation induced by amino acids.

(A) HEK293T cells were transiently transfected with plasmids for GFP (-) or GFP-TSC2 fragment (1717-1732 a.a.) (TSC2 frag) together with the plasmid for GST-HA tagged S6K1 (GST-HA-S6K1). After 24 h of transfection, cells were transferred to HHBSS for 60 min (−AA) and then stimulated with an amino acid mixture (+AA) for 30 min. Cell lysates were analyzed by Western blotting with indicated antibodies.

(B) Quantitation of the relative intensity of phospho-Thr389-S6K1 to total S6K1 of (A), in which the value of +AA in GFP-expressed cells was set to 1. Graphs represent mean ± SD of three independent experiments. One-way ANOVA with Tukey's test, ** $p < 0.01$, *** $p < 0.001$.

Legends for supplemental movies

Movies S1-S3

HeLa cells were transiently transfected with the plasmid encoding R-GECO1 (Movie S1), R-GECO-TSC2 WT peptide (Movie S2) or R-GECO1-TSC2 (WQLQ) peptide (Movie S3). The medium was replaced with HBSS for 15 min, and then ionomycin (2.5 μM) was added at 105 s to increase intracellular Ca^{2+} levels.

Movie S4

HEK293 cells transiently transfected with the plasmid encoding R-GECO1 were deprived of serum for 3 h, then carbachol (100 μM) was added at 120 s.
